# Value and Applicability of Large Administrative Healthcare Databases in Prosthetics and Orthotics Outcomes Research

**DOI:** 10.33137/cpoj.v4i2.35958

**Published:** 2021-09-21

**Authors:** TA. Miller, S. Wurdeman

**Affiliations:** 1 Department of Clinical and Scientific Affairs, Hanger Clinic, Austin, Texas, USA.; 2 College of Health and Human Services, University of North Carolina at Charlotte, Charlotte, North Carolina, USA.; 3 Department of Biomechanics, The University of Nebraska at Omaha, Omaha, Nebraska, USA.

**Keywords:** Health Economics, Prosthetics, Orthotics, Outcomes Research, Rehabilitation

## Abstract

The goal of health economics and outcomes research is to improve healthcare decision making. In the absence of high-value clinical data, the availability and quality of administrative healthcare data could be vital in the generation of evidence for orthotics and prosthetics services. The purpose of this article is to provide a stronger understanding of administrative healthcare data analysis, an area that has been scarcely examined within prosthetics and orthotics despite the wealth of information available within such data. Examples of common datasets in this arena currently available are provided, as well as an overview of the limitations and advantages of studies utilizing such datasets.

## INTRODUCTION

Health economics is a broad field of study with applications that focus on issues related to efficiency, effectiveness, equity, behavior and, ultimately, the value of healthcare services. The goal of health economics and outcomes research (HEOR) is to improve healthcare decision making for clinicians, managers, policy makers, payers, and patients. One aspect of HEOR that often comes to mind are the methods that assess cost and utilization (e.g. cost-effectiveness analysis or CEA). Understanding costs and utilization patterns can help define benefits of certain interventions, improve market access and establish the value of services or devices. However, additional areas of HEOR such as applications in health technology assessment, health service delivery and process of care, and patient-centered research can also lend themselves to better understanding the value of services or devices.^1,2^

Much of this data can be extracted and analyzed from administrative healthcare data. This paper is meant to provide a stronger understanding of administrative healthcare data analysis, an area that has been scarcely examined within orthotics and prosthetics (O&P) despite the wealth of information available within such data.

In the absence of high-value clinical data, the availability and quality of administrative healthcare data could be vital in the generation of evidence for O&P services. Several studies have assessed treatment interventions for those who require an ankle foot orthosis or a prosthesis, by using large databases in terms of epidemiology,^3,4^ clinical outcomes,^5^ and costs of treatment or utilization.^6^ Practical methods are needed by clinicians and researchers to address questions about the risks, benefits, and costs of interventions that inform the value of O&P health services. While randomized controlled trials (RCTs) remain a gold standard to establish efficacy and safety; they are not ideal for discovering effective or efficient treatment or for incorporation of the patient experience into clinical decision-making.^7^ Furthermore, conducting double blind RCT research is not always possible in O&P, which means our healthcare system lacks a comprehensive understanding of the incorporation of O&P devices into rehabilitation.

Real world evidence (RWE) studies, which capture effectiveness, and RCTs should be considered complementary to answer important healthcare questions. The use of prosthetic or orthotic devices aims to reduce the negative effects of disability (e.g. loss of work, isolation, and decreased independence) and alleviate burden on the healthcare system by improving treatment of conditions (e.g. stroke, amputation) that often require long-term rehabilitation or interaction with the healthcare system. Studies that use administrative health data are another way to generate information that contribute to the knowledge of O&P services.

Administrative data are real world data that can be leveraged to generate RWE as opposed to highly controlled and selective RCTs. Administrative data reflect the heterogeneous nature of populations. Specifically, claims data (i.e. billing data) or hospital discharge data (e.g. the Healthcare Cost and Utilization Project or HCUP) are typically referred to as secondary data sources because these data were not collected or generated for current research applications but rather for administrative use. While this may lead to some limitations in available information (e.g. limited functional data), they are often reported as reliable and consistent due to the nature and regulation of billing.^8^ It is worth noting though that the reliability of billing data is limited by the accuracy or integrity of the input. As more studies use administrative data, it highlights the increasing importance to be meticulous and aware of the data (e.g. diagnosis code, billing codes or L-codes) that are submitted for billing purposes.

A wide range of data elements comprise an administrative dataset. There are demographic variables (e.g. gender, race, age) and contextual factors (e.g. region of care, type of insurance), provider information (e.g. physician number), International Classification of Disease (ICD) diagnosis codes, Current Procedural Terminology (CPT), and Diagnosis-Related Group (DRG) codes. The year of the data dictates which diagnosis codes are used as ICD Ninth revision (ICD-9) was updated to the tenth revision (ICD-10) and implemented for billing in 2015. Due to this breadth of information, large administrative databases are appropriate and provide meaningful insight given the correct question, such as questions regarding national patterns of care or to determine resource expenditures. Yet, these large databases are not without limitations either.

## KEY CONSIDERATIONS AND APPLICATIONS

Administrative data are a unique source of information whose advantages and disadvantages for the scope of healthcare research have been extensively discussed.^8,9^ Briefly, a few limitations to be aware of are: conditions (diagnoses) must be diagnosed to be present, diagnosed conditions represent services provided but may not show potential need, conversely diagnosis codes not requiring services may not be recorded on a given claim, presence of a diagnosis does not inform on severity of the condition, and there is limited physiological data (i.e. blood pressure or functional mobility). Administrative databases are also often set up to be able to differentiate and characterize different lines of service within healthcare (e.g. services provided through an orthopedic surgeon versus vascular surgeon). Unfortunately, currently it is not possible to distinguish services provided by an orthotist/prosthetist versus another healthcare provider as O&P services are not differentiated from durable medical equipment for administrative data purposes.

However, there are advantages of studies that employ administrative data that can be leveraged through strong design methods. Advantages include validity of the data such as admission dates and procedure codes, systematic collection over time, cost data is reflected (charges or paid amounts), large samples allow for analysis of more rare conditions, and depending on the data it may be linked to other data sources (e.g. Medicare data can be linked to the national death index or national surveys).^8^ Because administrative health data contain large numbers of patients over long periods of time, as opposed to cross-sectional databases, they are useful to study disease associations with rare risk factors and heterogeneous populations. Specifically, in O&P, there is increasing demand for value-based evidence from studies that contain larger samples and information about the economic impact of treatment.^5,6,10^

When considering a research study using administrative data, in addition to the advantages and limitations discussed, it is also important to be aware of different characteristics of these databases. First, it is critical to consider the claims process. Health insurance claims data are based on information generated from the billing process for the purpose of payment when a patient utilizes health services. This includes inpatient services, outpatient services, emergency department visits, prescription drug utilization and laboratory utilization (e.g. blood work). Once the claim is reviewed by a payer, it is both accepted and paid, or not accepted. Therefore, coverage may vary from plan to plan and payer to payer. From the research perspective, often a claims dataset contains data that are adjudicated, meaning it is a complete set of data that represents services covered. For example, in prosthetics, if the office visit does not include a billable event (e.g. an alignment adjustment), then it will not be reflected in the data. However, while the patient maintains insurance coverage on a single plan (e.g. a Medicare beneficiary), it is possible to track the patient's journey from a hospitalization (i.e. inpatient services) to provision of a prosthesis or orthosis (i.e. outpatient services) and gain perspective on the overall utilization or pattern of care.

A second critical point to be aware of is the source of the claims. In general, there are two main sources in the US: commercial sources (e.g. private insurance) or non-commercial sources (e.g. Medicare or Veteran's Health Administration). It is important to consider your research question and identify the target population to answer the question. For O&P research, information gleaned from each of the different sources has the potential to inform different areas of care.

There are several different databases available that may provide good insight for O&P research, each with specific considerations depending on the research question (Table 1). These databases have varying requirements for access and cost. It is critical to have clear questions, transparency, and clarity in defining selection criteria when initiating a study. These decisions will help define which administrative databases will be the best match for the study purposes.

**Table 1:** Databases that contain prosthetic and orthotic services and/or patients that may require prosthetic or orthotic devices based on individual condition.

Dataset	Type of Data	Geographic area	Source	Access
Medicare	Billing claims of public program	National	Noncommercial	Must request access for patient level data but summary data may be public, fees vary
Medicaid	Billing claims public program for those with limited income and resources, includes nursing home care	State based, varies by state	Noncommercial	Must request access per state, fees vary
IBM Watson (Marketscan)	Billing claims for privately insured individuals, most often through employment	National	Commercial	Must request access, fees vary
IQVIA (Pharmetrics)	Billing claims for privately insured individuals, most often through employment	National	Commercial	Must request access, fees vary
Veteran's Health Administration	Department of Veteran's Affairs data, claims and aggregate data	National	Noncommercial	Some datasets are publicly available
Healthcare Cost & Utilization Project (HCUP)	A family of databases-discharge data	National and state	Contains data from both commercial and noncommercial	Most datasets are publicly available after completing online training, fees may apply

## CONCLUSION

In O&P rehabilitation care, we need to continue to be proactive in HEOR or risk further trailing behind other areas of healthcare. The way we as a field come together and agree upon how to capture, assess, and communicate value will determine our future as we differentiate ourselves from durable medical equipment. As we continue to be proactive in our investigations of HEOR we will be able to lead and enhance the assessment and value of O&P interventions, as well as demonstrate positive long-term outcomes for individuals who use O&P devices.

## CALL TO ACTION

Administrative data is, in general, a largely unexplored area of research for O&P. There are gaps in our knowledge regarding the health economic impact of O&P services and more evidence is needed on the effectiveness of O&P care. With increased reliance upon claims data from a payer and policy perspective, O&P clinicians and administrative persons need to be cognizant of an increased need for consistent coding across the profession. Clinicians and office administrators all play a role through daily work flow by enhancing quality control processes with regards to coding. Our industry organizations (e.g.: the American Orthotic and Prosthetic Association and the American Academy of Orthotists and Prosthetists) should take charge to implement standards and quality control processes surrounding coding to be adapted by professionals. The industry organizations should continue to work to separate orthotics and prosthetics from durable medical equipment as this will allow for cleaner claims analysis related to orthotic and prosthetic care. Orthotic and prosthetic schools should be teaching the role claims data can play in policy decisions to help drive the value of accurate and specific coding. To address gaps related to clinical practice guidelines and standards of care, communication between O&P providers, various specialists (e.g. surgeons, physiatrist) and physical therapists all involved in patient care is important to continue to optimize and standardize clinical practice protocols. Improved communication and active roles in billing and coding will in turn help produce effective and reliable databases for HEOR.

## DECLARATION OF CONFLICTING INTERESTS

The authors declare no conflict of interest.

## SOURCES OF SUPPORT

None.

## AUTHORS SCIENTIFIC BIOGRAPHY

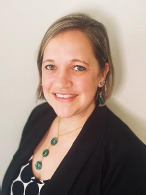



**Taavy Miller,** PhD, CPO, is a research scientist within Hanger's Department of Clinical and Scientific Affairs. Dr. Miller has broad experience working as a certified orthotist/prosthetist at large hospital-based systems and in private practice as well as teaching P&O at the university level. Dr. Miller holds a doctoral degree in Health Services Research with an emphasis in health economics and epidemiology. Her research focuses on health equity, reducing disparities and improving access through the assessment of health outcomes and effectiveness using administrative, clinical and patient reported data. She has published several studies in peer-reviewed journals and presented abstracts at national and international conferences.

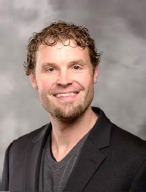



**Shane Wurdeman**, PhD, CP, is the Director of Clinical Research within Hanger's Department of Clinical and Scientific Affairs. He entered the field of O&P as a technician before transitioning to working as an orthotist/prosthetist and finally into his role as a principal investigator. Dr. Wurdeman holds a BS in physics, an MS in prosthetics and orthotics, and a PhD in biomechanics. He has co-authored more than 40 peer-reviewed manuscripts, published 3 book chapters, and presented more than 100 conference abstracts within the field of orthotic and prosthetic rehabilitation. He is a fellow with distinction of the American Academy of Orthotists and Prosthetists, from whom he was recognized in 2020 with their prestigious Academy Research Award. He currently serves as the Research Director for the American Orthotic and Prosthetic Association and Chair of the Center for Orthotic and Prosthetic Learning. He has been supported by private grants as well as government grants from the National Institutes of Health and Department of Defense.
